# A network-based integrative approach to prioritize reliable hits from multiple genome-wide RNAi screens in *Drosophila*

**DOI:** 10.1186/1471-2164-10-220

**Published:** 2009-05-12

**Authors:** Li Wang, Zhidong Tu, Fengzhu Sun

**Affiliations:** 1 Molecular and Computational Biology Program, University of Southern California, Los Angeles, CA 90089, USA; 2 Rosetta Inpharmatics, a wholly owned subsidiary of Merck & Co., Inc, 401 Terry Ave N, Seattle WA 98109, USA; 3 MOE Key Laboratory of Bioinformatics and Bioinformatics Division, TNLIST/Department of Automation, Tsinghua University, Beijing, PR China

## Abstract

**Background:**

The recently developed RNA interference (RNAi) technology has created an unprecedented opportunity which allows the function of individual genes in whole organisms or cell lines to be interrogated at genome-wide scale. However, multiple issues, such as off-target effects or low efficacies in knocking down certain genes, have produced RNAi screening results that are often noisy and that potentially yield both high rates of false positives and false negatives. Therefore, integrating RNAi screening results with other information, such as protein-protein interaction (PPI), may help to address these issues.

**Results:**

By analyzing 24 genome-wide RNAi screens interrogating various biological processes in *Drosophila*, we found that RNAi positive hits were significantly more connected to each other when analyzed within a protein-protein interaction network, as opposed to random cases, for nearly all screens. Based on this finding, we developed a network-based approach to identify false positives (FPs) and false negatives (FNs) in these screening results. This approach relied on a scoring function, which we termed NePhe, to integrate information obtained from both PPI network and RNAi screening results. Using a novel rank-based test, we compared the performance of different NePhe scoring functions and found that diffusion kernel-based methods generally outperformed others, such as direct neighbor-based methods. Using two genome-wide RNAi screens as examples, we validated our approach extensively from multiple aspects. We prioritized hits in the original screens that were more likely to be reproduced by the validation screen and recovered potential FNs whose involvements in the biological process were suggested by previous knowledge and mutant phenotypes. Finally, we demonstrated that the NePhe scoring system helped to biologically interpret RNAi results at the module level.

**Conclusion:**

By comprehensively analyzing multiple genome-wide RNAi screens, we conclude that network information can be effectively integrated with RNAi results to produce suggestive FPs and FNs, and to bring biological insight to the screening results.

## Background

In the past few years, many groups have successfully conducted multiple genome-wide RNA interference (RNAi) screenings in *C. elegans*, *D. melanogaster *and mammals, using either whole animal or cell lines to investigate a full array of biological processes at the systems level [1-4]. Compared with classical genetic screens, such as transposon-mediated mutagenesis and somatic clonal analysis [5-7], RNAi technology is revolutionary in that it allows investigators to quickly interrogate the phenotype changes that occur upon knocking down individual genes at the genome scale [8]. However, similar to many other high-throughput technologies, RNAi screens are not completely flawless. On the one hand, genes may not always be effectively knocked down and will consequently be missed by the screening. We refer to these genes as false negatives (FNs). On the other hand, owing to the tolerance for mismatches and gaps in base-paring with targets, small interfering RNA (siRNA) could possibly target up to hundreds of sequences [9,10], which are often termed as off-target effects (OTEs). Such OTEs are believed to be the main reason for false positives (FPs) in RNAi screens. The use of long double-stranded RNAs (dsRNAs) in *Drosophila *has been proposed as a means of reducing the occurrence of OTEs [11]. However, two groups reported that OTEs mediated by short homology stretches within long dsRNAs were prevalent in *Drosophila*, and that therefore the effectiveness of dsRNAs for reducing OTEs needs further investigation [12,13]. Furthermore, OTEs and low efficacies in knocking down certain genes are not the only sources for FNs and FPs associated with RNAi screens. As a matter of fact, designing a high-throughput RNAi screen involves many levels of decision-making, such as the type and concentration of RNAi reagents, the readout options, and the methodologies and criteria used for hit selections, each of which could affect the quality of the final results [11]. For example, it has been shown that the adoption of a better analytic method for hit selection may help reduce the rate of FPs and FNs [14-17].

Both computational and experimental efforts have been made to identify errors in RNAi screens. For example, Ma et al [12] and Kulkarni et al [13] suggested that dsRNAs which contained > = 19-nucleotide(nt) perfect matches to unintended targets or had simple tandem repeats of the tri-nucleotide CAN (N represents any base) might cause OTEs and thus contribute significantly to FPs. Consequently, sequence-based computational analysis can be used to predict potential FPs in RNAi screens. However, such prediction is not applicable to identifying FNs. Moreover, DasGupta and colleagues found that there was a lack of strict correlation between the sequence match of 19 nts and FPs, and they suggested that the "FP results" obtained from dsRNAs that were predicted to have OTEs based on sequence analysis should not be blindly treated as artifacts without further tests [18]. In their study, to experimentally distinguish true positives (TPs) from FPs, they rescreened hits identified in the original screen using multiple, independent "off-target (OT)-free" dsRNAs. However, such experimental validation has its own drawbacks. First, since not all dsRNAs are effective in knocking down the target genes, failure in validating the original positive hits is insufficient for validating FPs. In fact, they showed that some known regulators of the pathway under investigation were actually missed by the validation screens [18]. Second, since our knowledge of the mechanisms involved in OTEs is still developing, the successful validation of RNAi hits by so-called "OT-free" dsRNAs might actually be the result of unknown OTEs. Third, validation screens are usually conducted only on the positive hits from primary screens, and FNs cannot be recovered without additional effort.

As diverse genomic data accumulate, integrating RNAi screening results with other genomic information, particularly those represented in the form of networks, may help in identifying FPs and FNs. Network-based analysis has been widely applied to solving many biological problems. For example, methods have been developed using protein-protein interaction networks to predict unknown disease genes [19-22], or to diagnose disease subtypes [20]. A common principle adopted by most of these network-based studies is "guilt by association", i.e., nearby genes in the network are more likely to possess similar functions, or will lead to similar phenotypic changes, when perturbed. Here, we test whether this principle holds for RNAi hits, and if it does, we intend to apply it to addressing the noise issue associated with RNAi screens. We also anticipate that network analysis may help to reveal the underlying mechanisms that link the perturbed genes with the observed phenotype changes, which may not be directly obtainable from the raw screening data. Specifically, by perceiving the cell or organism as a dynamic system composed of interacting functional modules which are defined as discrete entities whose functions are separable from those of other modules [23], the network information can help us to identify the underlying module structure.

Here we present a comprehensive network analysis using 24 published genome-wide RNAi screens in *Drosophila*. We first verify the "guilt by association" principle by showing that RNAi hits are significantly more connected than random cases. We then develop a network-based RNAi phenotype scoring method termed NePhe to integrate information from both network topology and RNAi screening results. We demonstrate the effectiveness of NePhe scores in identifying putative FPs and FNs by a novel rank-based test and two case studies. We show how the network information can help identify the underlying modules as formed by the refined hits that potentially explain the RNAi phenotype changes as observed by the screen experiments. Finally, we discuss limitations of our approach and potential follow-up studies.

## Results

### RNAi hits have higher network connectivity than random chance hits

The *Drosophila *protein-protein interaction (PPI) network was built from PPIs in the STRING database [24]. STRING is a comprehensive PPI database, and the PPIs are experimentally derived or predicted by comparative genomics and text mining. In total, 10,297 proteins and 248,355 interactions were used to construct our network. Only proteins within this network were considered throughout our analyses. Hereinafter, we do not make explicit distinction between genes and their protein products.

24 genome-wide RNAi screening results [25-48] were downloaded from the flyRNAi database [49]. For each screen, we collected the set of genes that were observed to cause changes in the phenotype under investigation. We call these genes hits, and all the remaining genes nonhits for that screen. For each screen, a sub-network was constructed exclusively upon hits for that screen and the interactions among them. In order to evaluate network connectivity of these 24 sub-networks, we measured three network attributes, i.e., number of edges, size of the largest component and number of isolated nodes. We calculated two P-values for each attribute in each sub-network by either randomizing nodes or edges (see Methods for details). Table 1 lists the number of edges and P-values for each screen. In total, 20 out of 24 sub-networks have a significantly greater number of edges compared to randomized networks (both P-values < 0.005), supporting higher network connectivity. Similar, but slightly less significant, results were obtained for the other two network attributes (see Additional file 1 – Table S1). Therefore, our results indicate that the principle of "guilt by association" is valid and applicable for RNAi hits.

**Table 1:** The network attributes and corresponding P-values for the 24 sub-networks constructed from RNAi hits.

RNAi screen [Ref]	#hits	#edges	P-value1	P-value2
Store-operated Ca2+ entry [25]	1,122	4,281	2e-05	3e-98

ERK signaling [26]	982	7,050	2e-71	<1e-229

Nuclear import of Smads [27]	683	1,321	0.07	3e-06

Protein secretion and Golgi organization [28]	645	6,597	<1e-229	<1e-229

Hh signaling pathway [29]	306	3,214	<1e-229	<1e-229

Bacterial infection [30]	286	2,803	<1e-229	<1e-229

Growth and viability [31]	281	1,871	<1e-229	<1e-229

Wnt signaling pathway [32]	167	368	5e-51	1e-92

Light-dependent CRY degradation [33]	131	197	1e-28	9e-105

Neural outgrowth genes [34]	128	414	7e-146	3e-145

Chlamydia infection [35]	126	107	2e-07	2e-17

Regulators of NFAT [36]	121	29	0.7	0.002

Multipolar divisions [37]	115	62	0.005	9e-12

Viral replication [38]	104	2,069	<1e-229	<1e-229

JAK/STAT signaling [39]	104	85	4e-09	1e-21

Mycobacterial infection [40]	76	176	1e-129	8e-117

Transcript-specific mRNA export [41]	65	146	6e-141	2e-100

Ca(2+) influx [42]	65	137	3e-123	9e-54

Muscle assembly and maintenance [43]	39	21	5e-11	5e-08

Caspase activation [44]	37	4	0.4	0.2

Mitochondrial and Peroxisomal Fission [45]	22	2	0.3	0.1

Histone pre-mRNA processing [46]	17	4	2e-04	2e-05

E2F repression [47]	15	7	2e-13	1e-39

Orai proteins [48]	15	9	1e-21	1e-30

Results are sorted based on the number of hits in descending order. The P-value1 and P-value2 are calculated by two different randomization strategies, i.e., 1) randomizing nodes and 2) randomizing edges (with fixed node degree) (see Methods for details).

Although for most of the 24 screens, hits are significantly more connected than random cases, the degree of connectivity varies considerably among screens as reflected by the wide range of P-values. Several factors could account for this variance. For example, the STRING database may contain relatively more complete PPIs for some screened biological processes than others; therefore, some screening hits may appear to be more connected. Another possible factor could be the different accuracy for generating the screening results by different experimental protocols. For instance, as shown in Table 1, the sub-network constructed from "viral replication" [38] screening hits is among the most significantly connected, while the sub-network constructed from "nuclear import of Smads" [27] is among the least connected. Although the readouts were measured by immuno-fluorescence staining followed by automated microscopy for both screens, different accuracies could exist. In "viral replication", knockdowns of true participants were expected to cause a reduced number of cells compared to negative controls and thus presumably be easier to measure compared with the "nuclear import of Smads" process. In this case, the knockdowns were expected to cause diffused distribution of Mad in cytoplasm compared to the negative controls where Mad predominantly localized in nucleus, making it difficult to measure the phenotype change accurately and leading, in turn, to a higher error rate and lower connectivity. Furthermore, the criteria used for hit selection varied dramatically from screen to screen. For instance, as listed in Table 1, "Store-operated Ca2+ entry" [25] and "Ca(2+) influx" [42] are presumably two related biological processes. However, the two screens differ dramatically with regard to the number of hits and their associated P-values. The screen for "store-operated Ca2+ entry" measured the dsRNA effects by percentage inhibition and used a relatively lenient cutoff to obtain a large number of hits for further validation (1,122 hits). The screen for "Ca(2+) influx" calculated z-score for each dsRNA and used a relatively stringent cutoff of -3 to obtain a small number of hits (65 hits). The two hit sets overlap by 25 genes (Fisher Exact P = 5 × 10^-9^), suggesting that these two screens are significantly related, although very different in hit counts. Also screens are different as some kept the basic cell metabolism hits while some removed them. For instance, the sub-network associated with "JAK/STAT signaling" [39] appears to be less connected than that of the "Hh signaling pathway" [29]. What may partially account for this is the fact that the former removed ribosomal proteins, as well as proteins involved in RNA processing and translation during the curation process, while the latter did not. Finally, some of the hit sets listed in Table 1 were obtained directly from primary screenings, and some were filtered with additional validation assays. Although the 24 screening results studied here were curated from an assembly of experiments that varied in multiple aspects, the comprehensive study we performed here demonstrates that the higher network connectivity associated with RNAi hits and the applicability of our NePhe scoring system, as shown here and in later paragraphs, hold in general and are not restricted to a particular screening result.

### Identifying the best performing NePhe scoring system

Given the above observations, we then tried to apply the "guilt by association" principle to address the issue of FPs and FNs associated with RNAi screens. In general, we believe that if a gene has tight connection with many hit genes, then it is likely to be a TP in the case of a hit or an FN in the case of a nonhit, and vice versa. One computational problem that arises is the need to quantify the distance or similarity between a pair of genes in the context of network. Several different measurements have been proposed in previous studies to address similar problems [21,50,51]. We consider four of them: direct neighbor, shortest path, diffusion kernel [52] and association analysis-based transformation [51]. In addition, we also needed to quantify the overall similarity between a gene and its neighbors, or in an extreme case, all the remaining genes in the network. In this analysis, we considered three different summation formulas to calculate the overall similarity (see Methods for details). Thus, in total, we compared twelve different scoring functions, i.e., combinations of four pair-wise similarity measurements and three summation formulas (see Additional file 1 – Table S2). We call these scoring functions Network RNAi Phenotype (NePhe) scoring functions, since we integrate both the network topology and RNAi screen data to derive the NePhe scores.

**Figure 1:**
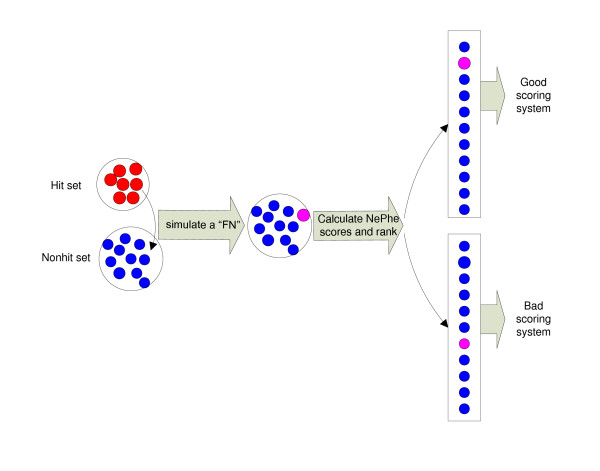
**The flowchart for the rank-based test**. We put one hit into the nonhit set as if it were a nonhit (simulated FN). We then ranked all nonhits, including the simulated FN, using different scoring methods. Presumably, a good scoring system can rank the "FN" higher, while a bad scoring system cannot.

Since full annotations for true positive hits or true negative hits are not available for most RNAi screens, it is not possible to directly compare the performance of each scoring method. To overcome this difficulty, we designed a rank-based test to indirectly estimate the relative performance of different scoring functions (see Methods for details). Although the NePhe scoring functions differ in how they define pair-wise similarity and how to summarize the similarity across all neighbors, the common scenario is that a gene would receive a higher score if a greater number of its neighbors are hits. Therefore, under the principle of "guilt by association", an FP should be more likely to receive a lower score compared to TPs. In contrast, an FN should be more likely to receive a higher score compared to TNs. Based on this reasoning, the rank-based test works as indicated in the following description. We assume that all hits in the original RNAi screens are TPs and all nonhits are TNs. One hit is placed into the nonhit set as if it were a nonhit (simulated FN). We then rank all nonhits, including the simulated FN, using different scoring methods (see Figure 1). Similarly, one nonhit is added to the hit set as though it were a hit (simulated FP), and we then rank all hits, including the simulated FP, using different scoring methods. We repeat the above procedure for each hit and nonhit for each screen. We evaluate the performance of each scoring method based on two quantities: the relative rank (RR) of simulated FNs among nonhits and the RR of simulated FPs among hits. For each scoring function, we calculate the group means of the two quantities for each screen, and the overall performance of each method is determined by the grand means of the two quantities from all 24 screens (see Methods for details). Theoretically, for an optimal scoring method, the RR of FNs should be close to 1 (ranked at the top), and the RR of FPs should be close to zero (ranked at the bottom). In reality, however, because not all hits in the original hit sets are TPs, we do not expect every FN simulated in this way to be ranked high among all negatives, as not all negatives are TNs either. Similarly, we do not expect every simulated FP to receive a low rank among all positives. However, as long as the original hit sets are significantly enriched for TPs (which we believe to be true for most screens), the rank-based test should reflect the relative performance of each method.

**Figure 2:**
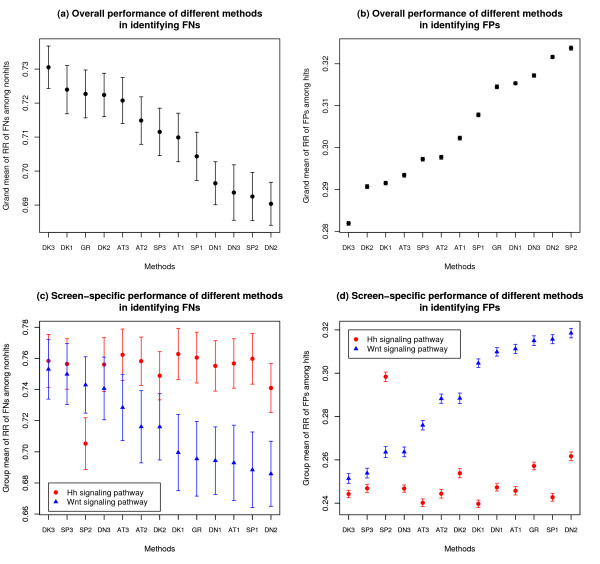
**The overall performance of different methods in identifying FNs (a) and FPs (b) in the rank-based test and the screen-specific performance of different methods in identifying FNs (c) and FPs (d) in the rank-based test**. The error bars represent the estimated standard deviations for the corresponding quantities. The DN, SP, DK and AT represent the four different network similarity measurements, i.e., direct neighbor, shortest path, diffusion kernel and association analysis-based transformation, respectively. Index 1, 2 and 3 represent the three different summarizing formulas, respectively (see Additional file 1 – Table S2 for details). GR represents the GeneRank algorithm.

Figure 2 shows the performance of different NePhe scoring methods estimated by the rank-based test. First, as indicated by the grand mean of FPs and FNs, all the network-based scoring methods perform much better than random chance (which is expected to be 0.5) in ranking FPs and FNs for all 24 screens (Figure 2(a) and 2(b), respectively). It is of note that the error bars, which represent the standard deviations, are considerably larger in Figure 2(a) than in Figure 2(b). This is expected since there are fewer hits than nonhits for all the screens, hence fewer simulated FNs than FPs. Second, when considering the overall performance of the four similarity measurements, diffusion kernel performs the best, followed by association analysis-based transformation, shortest path, and then direct neighbor. This result is consistent with previous studies [21,50] and supports the superiority of global measurements (e.g., diffusion kernel) over local measurements (e.g., direct neighbor). When considering the three summation functions, formula 3 performs slightly better than the other two. However, the summation formulas have less influence on the overall performance compared to similarity measurements. The better performing formula 3 endorses the calculation of a gene's similarity to other genes by putting different weights to hits and nonhits (see Methods). Third, when we compare the NePhe scoring system with the GeneRank algorithm [53], we find that GeneRank is not as powerful as our models in recovering FPs (Figure 2(b)), but is comparable in prioritizing FNs (Figure 2(a)). We optimized the parameter *d *in the GeneRank algorithm by varying it from 0.1 to 0.9 with 0.1 intervals, and selected the one with the best performance to compare with our best performed models. Finally, there is no consistently best performing NePhe scoring function, and the best function is somehow screen-specific. Figure 2(c) and Figure 2(d) show the screen-specific performance of different methods for two screens that are used for case studies in later sections (see Additional file 1 – Figure S1 for all the 24 screens). It should be noted that the relative performance of different methods varies considerably for each screen. One possible reason for this is that different RNAi hit sub-networks may have distinct characteristics. For example, hit sub-networks of smaller size appear to favor the diffusion kernel method over the direct neighbor method (see Additional file 1 for details). Since the rank-based test can tell us which method is the best for a particular screen, we simply use the method with the best performance throughout our analyses with the following two case studies.

**Figure 3:**
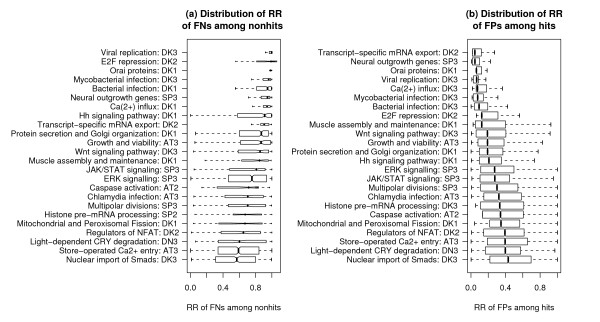
**The distributions of RR of FNs among nonhits (a) and RR of FPs among hits (b) for each screen in the rank-based test**. The RR was computed by the best performing scoring method for that screen according to the rank-based test. The notation for each method is the same as in Figure 2.

To further quantify the RR of simulated FNs among nonhits and the RR of simulated FPs among hits, we show their distributions for each screen in Figure 3. As seen in Figure 3(a), 14/24 screens have their majority (>50%) of simulated FNs ranked above 0.8 among all negatives, or 9/24 screens, if considering a higher threshold of 0.9. Similarly, 15/24 screens have their majority of simulated FPs ranked below 0.3 among all positives, or 7/24 screens, if considering a lower threshold of 0.1 (Figure 3(b)). Furthermore, we find that the results shown in Figure 3 correlate well with results in Table 1, i.e., the effectiveness of the NePhe scoring system for a particular screen largely correlates with the degree of connectivity of the sub-network derived from that screen. The two related screens, Ca2+ influx and store-operated Ca2+ entry, received quite different ranks among all the 24 screens based on RR of FNs and RR of FPs. Since the screen for Ca2+ influx used a more stringent cutoff to call hits, the number of hits is much smaller (65 hits) compared to the screen for store-operated Ca2+ entry (1,122 hits) and is presumably, therefore, of better quality. The better rank it received based on the NePhe scoring system suggests that the output of our approach is reasonably dependent on the quality of its input. For the two screens that we use for later case studies, i.e., "Hh signaling pathway" and "Wnt signaling pathway", the performance of NePhe scoring is intermediate among all 24 screens.

### Case studies: Hedgehog (Hh) and Wnt signaling pathways

In this section, we study RNAi screens interrogating Hh [29] and Wnt signaling pathways [32]. Because all the original hits had been rescreened by an independent collection of dsRNA to assess FP rates in a follow-up study [18], we chose these two particular screens. Thus, for these two particular RNAi screens, we can use this validation screen as an independent experimentally derived reference set to estimate the performance of the NePhe scoring system in identifying FPs.

### Comparing NePhe scoring system with experimental validation

We compare the NePhe scoring system with experimental validation from the following four aspects.

**Figure 4:**
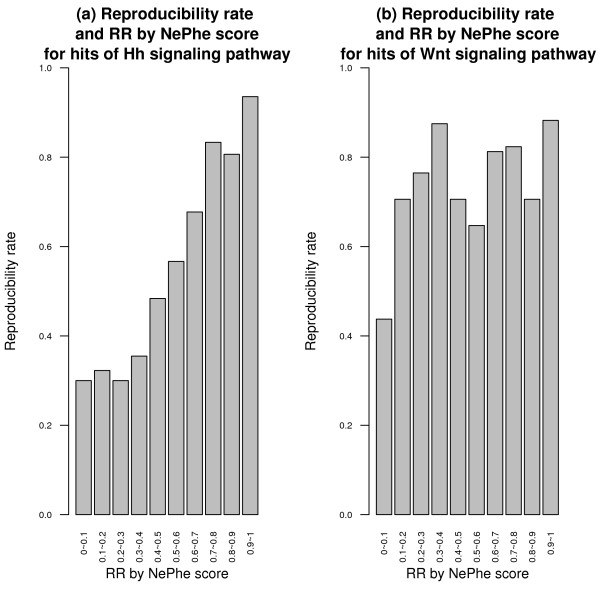
**The reproducibility rate of hits in the validation screen within each interval of the RR by NePhe score for Hh (a) and Wnt (b) signaling pathway**.

First, NePhe scores correlate with experimental validation results. We ranked hits in the original screen of Hh signaling pathway by their NePhe scores and put them into bins. Within each interval, we calculated the proportion of hits confirmed by experimental validation, termed as the reproducibility rate. As shown in Figure 4(a), the reproducibility rates positively correlate with the NePhe scores. Statistical tests show that the ranks of all reproducible hits are significantly higher than those of irreproducible hits (P-value = 4e-14 by Wilcoxon rank-sum test). A similar, but weaker, trend can be seen for the Wnt signaling pathway (P-value = 0.04 by Wilcoxon rank-sum test) (Figure 4(b)). The decrease of reproducibility rates is most visible for RR between 0 and 0.1, but less apparent for other intervals. One possible reason is that the original hit set for the Wnt signaling pathway was already quite accurate. In fact, the overall reproducibility rate for the Wnt signaling pathway is about 74%, higher than that for the Hh signaling pathway (56%). As the validation experiment is also based on RNAi technology, it has its own FP/FN issues and may fail to validate an already very accurate hit set. Here, the 74% reproducibility rate is comparable to the average reproducibility rate observed for the validation screen when the same collection of dsRNA was used to self-validate, which clearly demonstrates a limitation of experimental validation [26].

Second, the NePhe scoring system can prioritize known regulators of Hh/Wnt pathways that failed to be confirmed by experimental validation. As discussed in the introduction, validation experiments are based on RNAi and can have their own FN issues. Using Hh and Wnt signaling pathways as examples, we find that the validation experiment indeed failed to validate some of the known pathway regulators. As shown in Table 2, the KEGG [54] pathways contain 25 and 65 genes for Hh and Wnt signaling pathways, respectively (we regard them as true positives). The original screens identified 9 and 9 of these known regulators, respectively (Table 2). However, the validation experiment only confirmed 6 out of 9 for the Hh signaling pathway and 8 out of 9 for the Wnt signaling pathway. Those unconfirmed regulators may be suggestive FNs of validation screens, although they could also be missed by the validation screens for a multitude of reasons unrelated to the FN/FP rate (see the discussion). On the other hand, the NePhe scoring system seems to successfully capture all the known regulators in the original hit sets. By calculating the NePhe scores for all the original hits (306 and 167 for Hh and Wnt signaling pathways, respectively) and choosing the top ranked hits of the same size as the experimentally validated hit sets (171 out of 306 and 123 out of 167), we see that all the original hits contained in KEGG pathways are kept in these NePhe top-ranked hit sets (Table 2). Therefore, compared with the validation experiments, the NePhe scoring system is better at keeping hits that are known regulators, while some of these hits are missed by validation experiments.

**Table 2:** The overlap between KEGG pathway genes and hits/nonhits in the corresponding RNAi screen.

KEGG pathway (#genes)	All hits	Experimentally validated hits	Top-ranked hits	All nonhits	Top-ranked nonhits (RR > 0.9)	Top-ranked nonhits (RR > 0.8)
Hh signaling pathway (23)	9	6	9	14	7	10

Wnt signaling pathway (65)	9	8	9	56	32	48

Each cell represents the overlap between KEGG pathway genes (rows) and all, or subsets of, hits/nonhits in the corresponding RNAi screen (columns). The hits/nonhits were ranked by NePhe score. The top-ranked hits (column 3) are of the same size as the experimentally validated hits (column 2).

**Figure 5:**
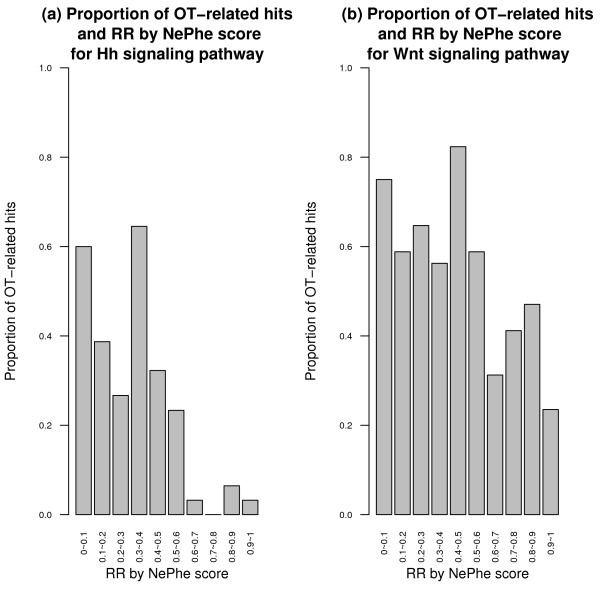
**The proportion of OT-related hits within each interval of the RR by NePhe score for Hh (a) and Wnt (b) signaling pathway**.

Third, NePhe scores correlate with sequence-based OTE prediction for FPs. As discussed in the introduction, OTEs mediated by homologous sequences or CAN repeats are believed to be a main reason for RNAi screen FPs. We classified screening hits into two categories, i.e., off-target (OT)-related and OT-unrelated, using sequence-based OTE prediction similar to DasGupta *et al. *[18] (see Additional file 1 for details). Figure 5(a) shows the proportion of OT-related hits for each NePhe score interval. It can be seen that there is a strong negative correlation between the proportion of OT-related hits and their RR for both the Hh signaling pathway and Wnt signaling pathway. Statistical tests show that the rank of OT-related hits is significantly lower than that of OT-unrelated hits (*P *= 4e-13 for the Hh signaling pathway, and *P *= 9e-4 for the Wnt signaling pathway by Wilcoxon rank-sum test). Therefore, FPs predicted from the NePhe score correlate well with those predicted using sequence-based OTE prediction.

**Figure 6:**
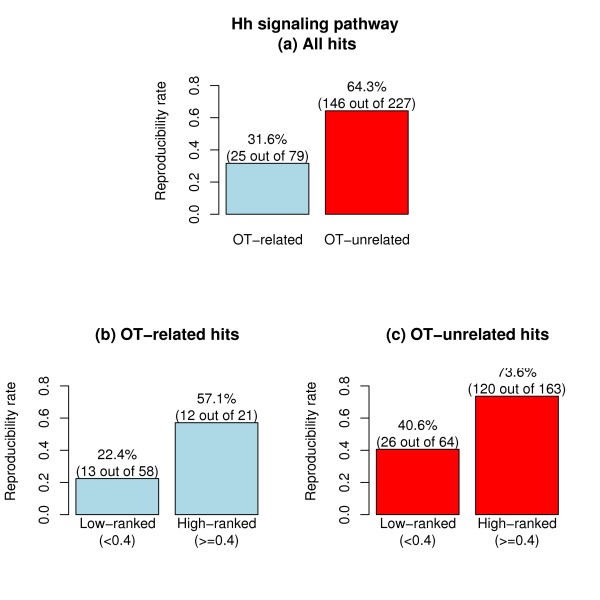
**The reproducibility rate for OT-related and OT-unrelated hits (a), for low-ranked and high-ranked OT-related hits by NePhe score (b) and for low-ranked and high-ranked OT-unrelated hits by NePhe scores (c) for Hh signaling pathway**.

Fourth, the NePhe score can further refine the sequence-based OTE prediction for FPs. DasGupta and colleagues pointed out that there was a lack of strict correlation between predicted OT-related hits and FPs as confirmed by validation experiments [18]. Figure 6(a) shows the reproducibility rates for OT-related and OT-unrelated hits based on the validation experiment for the Hh signaling pathway. It is clear that OT-related hits have a lower reproducibility rate, indicating that sequence-based OTE prediction is generally informative. However, 31.6% of OT-related hits were in fact reproduced in the validation screen, indicating that FPs predicted by sequence analysis could actually contain a considerable proportion of TPs. Similarly, TPs predicted by sequence analysis could in fact be contaminated by a considerable proportion of FPs. Using the NePhe scores, we further divided OT-related and OT-unrelated groups into high-ranked (e.g., RR of NePhe score > = 0.4) and low-ranked subgroups. Here, the cutoff of 0.4 is somehow arbitrary, but we get similar results using different cutoffs from a reasonable interval (data not shown). We compute the reproducibility rate for the four subgroups separately. The results are plotted in Figure 6(b) and 6(c), and it can be seen that the high-ranked subgroup has a much larger reproducibility rate than the low-ranked subgroup for both OT-related and OT-unrelated hits. Statistical tests further confirm that the rank of reproducible hits is significantly higher than that of irreproducible hits for OT-related and OT-unrelated hits (Wilcoxon rank-sum test *P *= 0.04 for the former and 1e-9 for the latter). A similar, but less significant, pattern is also observed for the Wnt signaling pathway (see Additional file 1 – Figure S2). Therefore, the NePhe scoring system can be used to further identify TPs from predicted FPs (OT-related hits) or FPs from predicted TPs (OT-unrelated hits).

### Identifying FNs from nonhit set using NePhe scoring system

Compared to experimental validation, the value of the NePhe scoring system becomes clearer when we consider recovering FNs that are missed by the original screens. This is because in practice, most experimental validations focus only on primary hits, as does the sequence-based OTE prediction. In this subsection, we provide evidence indicating that top-ranked nonhits by the NePhe scoring system are enriched for genes that are relevant to the pathway under investigation, while these nonhits are putative FNs missed by the original screens.

First, top-ranked nonhits are enriched for known regulators of the Hh and Wnt signaling pathways. Table 2 lists the numbers of top-ranked nonhits that are known KEGG pathway genes. For each pathway, 14 out of 56 KEGG pathway genes were missed by the original screens and thus reported as nonhits. By ranking all the nonhits from original screens based on their NePhe scores, we see that the top 10% nonhits were able to capture 50% (Hh) and 57.1% (Wnt) of these missed KEGG pathway genes (P-values are 1e-4 and 2e-18 by Fisher Exact Test). The top 20% nonhits are able to capture 71.4% and 85.7% of these missed KEGG pathway genes (P-values are 4e-5 and 4e-26 by Fisher Exact Test).

**Figure 7:**
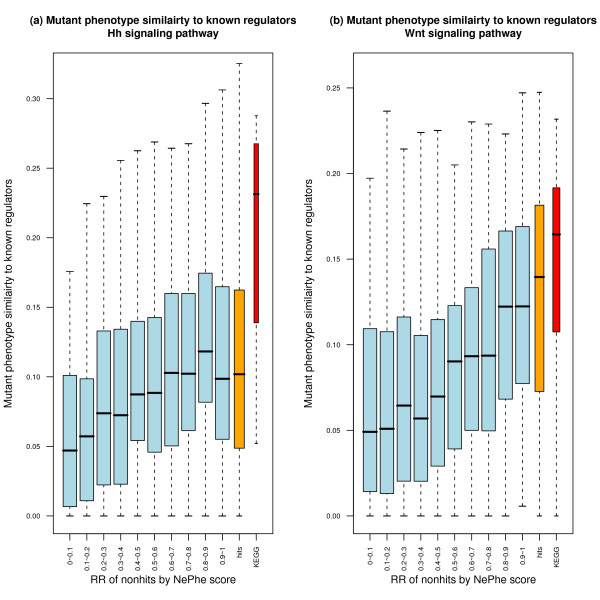
**Distributions of the mutant phenotype similarities between nonhits within each interval of the RR by NePhe score and known regulators (blue bars), between hits and known regulators (orange bar) and among known regulators themselves (red bar) for Hh signaling pathway (a) and Wnt signaling pathway (b)**. Only genes with at least one allele phenotype are considered here.

Second, top-ranked nonhits display mutant phenotypes similar to the mutant phenotypes for known Hh or Wnt pathway genes. Here we assume that genes belonging to the same pathway tend to show similar phenotypes when mutated. Because we have a set of known regulators from KEGG, we can compare the mutant phenotypes to estimate how likely it is that an unknown gene belongs to the same pathway. We retrieved allele phenotype data for *Drosophila *genes from FlyMine [55] and used these data for mutant phenotypes. There were 1,901 genes that had at least one allele phenotype, and we only considered them in the following analysis. We calculated the mutant phenotype similarity between each nonhit and known regulators (see Methods for details). The distributions of the similarities for nonhits with different NePhe scores are shown in Figure 7 in blue bars. We also computed the similarity between each hit and known regulators (orange bar in Figure 7), and the phenotype similarity among known regulators (red bar in Figure 7). First, as expected, the within pathway mutant phenotype similarity is the highest for both Hh and Wnt KEGG pathways, which supported our assumption. Second, there is a significant positive correlation between the RR of nonhits and their mutant phenotype similarities to known regulators (P-value < 2e-16 by Spearman's correlation test; estimated rhos are 0.28 and 0.31 for the Hg and Wnt pathways, respectively). The strong correlation observed here indicates that the NePhe scoring system indeed correctly ranked putative FNs to the top of nonhit genes. Third, with regard to mutant phenotype similarity to known regulators, there is no significant difference between top-ranked nonhits (rank >0.9) and hits (P-value > 0.1 by Wilcoxon rank-sum test). In other words, these top-ranked nonhits are comparable to hits when mutant phenotype similarity to known regulators is considered. The failure of RNAi screens to detect these putative FNs might result from ineffective knockdown by siRNA. Or, it could also result from the fact that the RNAi screens were carried in cell lines and thus unable to capture certain regulators with detectable mutant phenotypes only at the tissue or organism level. In any case, the NePhe scoring system can be used to identify putative FNs that are not identifiable by experiment alone.

### Interpretation of RNAi phenotypes at module level

**Figure 8:**
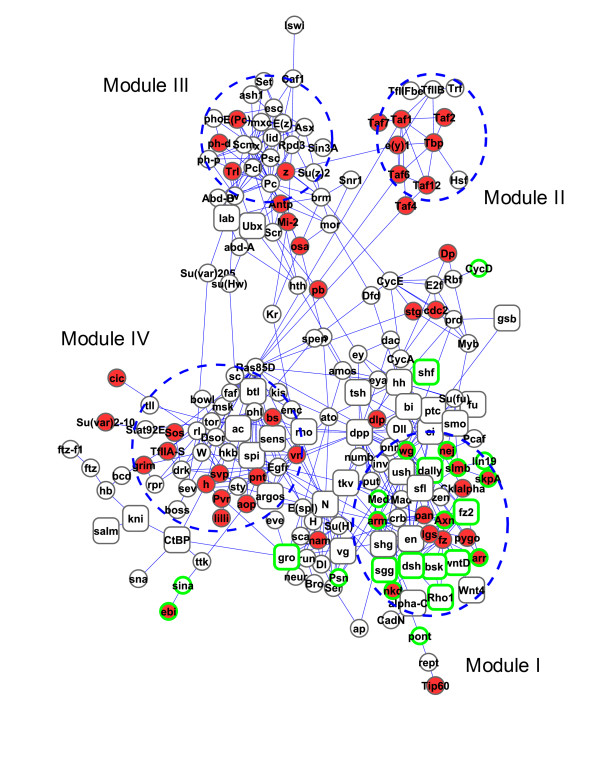
**A sub-network associated with the Wnt signaling pathway**. Red: hits of RNAi screening. White: top-ranking nonhits by NePhe score. Green boundary: genes within KEGG Wnt signaling pathway. Square: genes supported by literature for their association with the Wnt pathway. Module I: canonical participants-related. Module II: transcription factor TFIID complex-related. Module III: PcG protein complex-related. Module IV: other signaling pathways-related. The visualization was generated using Cytoscape [57].

In this section, we use the Wnt signaling pathway as an example to show how the NePhe scoring system can bring biological insights to the screening results and can help to interpret RNAi phenotypes at the module level. Based on the RNAi screening results and NePhe scores, we constructed a high-confidence Wnt signaling pathway-related sub-network. The sub-network was built by hits in the original screen, top-ranked nonhits by NePhe score and high-confidence interactions in the STRING network (confidence score >0.9 [56]). We included the top 300 nonhits to the sub-network for two reasons: 1) the RR is high, >0.97, and 2) its accuracy is most likely comparable to the original screening hit set using the KEGG Wnt signaling pathway as reference because both sets contain a similar proportion of Wnt KEGG pathway genes (~5%). Figure 8 (generated using Cytoscape [57]) shows the largest connected component of this sub-network consisting of 209 genes in total, among which 51 are hits (red), 158 are top-ranked nonhits (white), 24 are members in the KEGG Wnt signaling pathway (green boundary), and 41 are supported by literature for their association with the Wnt signaling pathway (square). We refer hereafter to those genes within the KEGG Wnt signaling pathway as canonical participants. 15 out of the 24 canonical participants shown in Figure 8 are among the top-ranked nonhits (e.g., *dsh, dally*), which further confirms the effectiveness of our computational strategy in recovering putative FNs of RNAi screens. What might be more interesting in Figure 8 is that, with the network information and the nonhits recovered by NePhe scores, hits detected in the RNAi screen appear to be clustered into several hypothetical modules. These modular structures may help us to dissect the potential roles of module genes, including the non-canonical participants, in the Wnt signaling pathway.

Examination of these hypothetical modules and their functions indicates that the Wnt signaling pathway could be regulated at different levels with varied specificities (see Additional file 1 for details). From the most general regulators, such as TFIID complex, to less general regulators that are preferentially involved in controlling signaling pathways (e.g., PcG complex), to participants of other signaling pathways that enable cross-talkings, and to the most specific regulators like the canonical participants of Wnt signaling pathway, the network approach we developed just reveals a fine modular view of the Wnt signaling pathway that could be of great interest to biologists for further validation, and such views are not directly derivable from raw RNAi screen data.

## Discussion and Conclusion

We carried out by far the most comprehensive network-based analyses on multiple genome-wide RNAi screens in *Drosophila*. We showed that RNAi screen hits were generally more connected in the PPI network than random cases. We developed a NePhe scoring system to identify both FPs and FNs in RNAi screening results. We demonstrated the power of such scoring system by a novel rank-based test and two case studies. We provided our NePhe score results for both hits and nonhits of the 24 whole-genome RNAi screens to provide a foundation for follow-up studies (see Additional file 2). We also showed that these NePhe scores are reasonably robust to the random noise in the initial hit sets (see the Additional file 1 for details). We implemented our strategy to compute NePhe scores in an R package (see Additional file 3) so that our approach could be used by the whole research community in the future.

With that said, our approach does have several limitations. First, the NePhe scoring system relies on the relative completeness and accuracy of PPI information. It is not applicable to hits or nonhits whose target genes are missing from the interaction network. Likewise, genes present in the network, but which have poorly characterized interactions, are likely to yield inaccurate results. Second, the list of putative FNs and FPs created by our approach are only suggestive and should not be regarded as definitive. Although hits with lower NePhe scores will most likely validate at lower rates than those with higher scores, the exact fraction of them representing true FNs and FPs cannot be known without actually validating the data. For example, even though a gene is relevant to a pathway under investigation, its knockdown does not need to have an effect for a multitude of reasons, such as the paralogue issue and the alternative/branching of pathway issue. Thus, some putative FNs suggested by our approach may not necessarily be FNs. In this regard, scientists should use our approach to assess the general robustness of their screen rather than use it as a substitute for experimental validation. Third, our approach does not address the "specificity" issue with RNAi screens. A common phenomenon observed by many screens is that genes related to basic cell metabolism (e.g., ribosomal proteins, proteasome components, polymerases, or splicing factors) are often reported as hits. These genes usually receive high NePhe scores since they are well connected in the network. Although they are likely to be true hits, they may not be relevant to the questions being asked of each screen. Some of these effects could be offset by cross comparing multiple screening results. For example, we can remove from each screen those genes that participate in cell growth and viability [31] and perform the NePhe calculation afterwards (see Additional file 1 – Table S3 for the network connectivity of each RNAi screen after the removal). However, such strategy will miss bona fide components that may have pleiotropic effects [8,58].

In summary, we present a novel network-based strategy that can potentially address the FN and FP issue associated with RNAi screens. Follow-up experimental validations of our results are extremely valuable for further quantifying the results of our approach. Moreover, given the increasing popularity of RNAi techniques and rapidly accumulating protein-protein interaction data in multiple model organisms, including human, the applicability of our approach to other species is very promising.

## Methods

### Network attributes, P-values and Randomization

24 RNAi screens were considered in total, and each screen was analyzed independently. For each screen, a sub-network was constructed by including only hits for that screen and PPIs among them as obtained from STRING database. We calculated three network attributes, i.e., number of edges, size of the largest component and number of isolated nodes. In order to obtain P-values for each network attribute, we constructed randomized sub-networks for each of the 24 RNAi screens. From these randomized sub-networks, we obtained null distribution of each network attribute for that particular screen. The P-value was then computed by one-side testing assuming these attributes under null hypothesis were normally distributed, while mean and variance were estimated from these randomized sub-networks. The assumption of normal distribution has been verified and adopted in a previous study in yeast [59]. We employed two different strategies in generating randomized sub-networks from the whole PPI network. In the first strategy, we randomly assigned a gene in the whole network as a hit while keeping the total number of hits the same as seen from the real screen. Then we derived a sub-network associated with the randomized hits from the whole network. In the second strategy, we first randomized the edges of whole network while keeping the number of interactions for each node fixed (implemented by R graph package). From the randomized whole network, we then derived a sub-network associated with the original hits. For each strategy, we generated 1,000 randomized sub-networks.

### Network-based similarity

The network-based similarity *S*_*ij *_between gene *i *and *j *were measured in the following four ways.

#### Direct neighbor

(1.1)

where A is the adjacency matrix, so that .

#### Shortest path

(1.2)

where *sp*_*ij *_denotes the shortest path between *i *and *j *in the network. We used the exponential function *y *= exp(-*x*) to transform the gene-gene distance to gene-gene similarity.

#### Association analysis-based transformation

(1.3)

where *d*_*i *_denotes the degree of node *i *in the network. It is known that PPI networks are both incomplete and inaccurate. One way to handle this problem is to transform the original interaction graph to new graphs by removing spurious edges, adding biologically valid ones, and assigning reliability scores to the edges constituting the final network [51]. The motivation for the above transformation method is that proteins sharing many neighbors are more similar, and the significance of this similarity depends on the number of neighbors that each gene has.

#### Diffusion kernel

(1.4)

where *K *= exp (β*L*), *L *= *A *- *D*, and *A *form the adjacency matrix of the interaction network and *D *is a diagonal matrix containing the nodes' degrees. The diffusion kernel can be seen as a random walk consisting of transitions to each one of the current node's neighbors with probability of β [52]. *K*_*ij *_can be regarded as a sum of the probabilities over all paths from *i *to *j*. In this study, we explored two different values forβ, i.e., 0.1 and 0.01, and chose the one that gave better performance in the rank-based test.

### NePhe scoring system

The NePhe score of gene *j *for screen *k *is calculated by the following three formulas:

(2.1)

(2.2)

(2.3)

where *G *denotes all the genes in the network, *S*_*ij *_denotes the network similarity between gene *i *and *j*, and *I*_*ki *_denotes the observation of gene *i *in RNAi screen *k*. *I*_*ki *_= 1 if gene *i *is a hit for screen *k*, and 0 otherwise.

Intuitively, the more similar a gene is to hits according to the network-based similarity (Formula 1.1–1.4), the more likely it is a TP/FN. Thus, a straightforward scoring function would summarize the similarity of gene *j *to all hits of the screen *k *(Formula 2.1). However, the similarity of a gene to nonhits may also affect its likelihood of being a TP/FN, i.e., higher similarity to nonhits may indicate lower possibility of being a TP/FN. Motivated by this, we devised Formula 2.2 which divides the similarity of gene *j *to all hits by its similarity to all the genes (both hits and nonhits). In order to distinguish the different contributions of hits and nonhits to the final score, Formula 2.3 combines the similarity of gene *j *to all hits and all nonhits with different weights, i.e., α_*k *_and β_*k*_.

In order to determine the parameters α_*k *_and β_*k *_in Formula 2.3, we used the following linear regression model:

(2.4)

Given observations for all genes {*I*_*kj *_| *j *∈ *G*} in screen *k*, the above linear regression optimizes the coefficients γ_*k*_, α_*k *_and β_*k*_so that the model, which differs from the NePhe score (Formula 2.3) by a constant γ_*k*_, would predict actual observations with the least square errors. Here, we adopted linear regression instead of logistic regression since the former model performed slightly better than the latter one in rank-based test (see Additional file 1 for details). However, as linear model assumes the normality of the residual variables, alternative models which make no such assumptions could lead to better performance.

Different from previous studies[53,60], the network-based similarity of a gene to itself is not considered in any of the above three formulas (2.1–2.3). In other words, the NePhe scoring functions do not consider a gene's own RNAi phenotype in the screen. This is particularly useful when we need to predict a gene's knockdown phenotype type which has not been previously screened by RNAi.

### Rank-based test

To systematically evaluate the performance of the above different scoring methods in identifying FNs and FPs in the RNAi screening results, we designed a novel rank-based test.

We simulated FNs by adding one hit gene into the nonhit set of screen *k *each time. Specifically, for a gene *i *∈ {*i *| *I*_*ki *_= 1} (*I*_*ki *_specifies if gene *i *is a hit for screen *k*), we set *I*_*ki *_= 0 and considered it as an FN. We updated the NePhe score according to Formula 2.1–2.3 for each gene with current setting of *I*_*ki*_. Particularly, to mimic real situations, we also updated the parameters of α_*k *_and β_*k *_in Formula 2.3 by linear regression with current setting of *I*_*ki*_. We then ranked all genes in the nonhit set by NePhe score, which includes all the original nonhits and the simulated FN gene *i*. We denote the RR of the simulated FN gene *i *as *FNR*_*ki *_(*FNR*_*ki *_∈ (0,1]). We set *I*_*ki *_back to 1 afterwards. We repeated the above procedure for each gene *i *∈ {*i *| *I*_*ki *_= 1}. The screen-specific performance of a scoring method in identifying FNs for screen *k *is defined as the group mean of *FNR*_*ki*_:

(3.1)

The overall performance of a scoring method in identifying FNs for all the 24 screens is defined as the grand mean of *FNR*_*ki*_, which is the mean of the group mean of *FNR*_*ki*_.

(3.2)

where *E *denotes the set of all 24 screens. If a phenotypic score performs well in recovering FNs, we would expect  and  close to 1.

Similarly, we simulated FPs by adding one nonhit gene into the hit set of screen *k *each time. Specifically, for a gene *i *∈ {*i *| *I*_*ki *_= 0}, we set *I*_*ki *_= 1 and considered it as an FN. We updated the NePhe score for each gene with current setting of *I*_*ki*_. We then ranked all genes in the hit set by NePhe score, which includes all the original hits and the simulated FP gene *i*. We denote the RR of the simulated FN gene *i *as *FPR*_*ki *_(*FPR*_*ki *_∈ (0,1]). We set *I*_*ki *_back to 0 afterwards. We repeated the above procedure for each gene *i *∈ {*i *| *I*_*ki *_= 0} The screen-specific performance of a scoring method in identifying FPs for screen *k *is defined as the group mean of *FPR*_*ki*_

(3.3)

The overall performance of a scoring method in identifying FNs for all 24 screens is defined as the grand mean of *FNR*_*ki *_which is the mean of the group mean of *FNR*_*ki*_.

(3.4)

where *E *denotes the set of all 24 screens. If a phenotypic score performs well in recovering FNs, we would expect  and  close to 0.

### Mutant phenotype similarity

We downloaded allele phenotype data for *Drosophila *from FlyMine [55]. In specific, for each gene, we obtained a list of distinct terms describing the phenotypes that had been manifested by at least one of its alleles, including the tissue type, cell type, and developmental stage. After mapping to the PPI network, we obtained 1,901 genes associated with at least one of the total 2,884 distinct phenotype terms. We represented the phenotype profile for each gene as a Boolean vector of length 2,884, specifying whether it was associated with each of the 2,884 phenotype terms. The mutant phenotype similarity between two genes was then calculated by the cosine of the angle between their phenotype vectors as in the previous study [61]:

(4.1)

The mutant phenotype similarity of a gene to KEGG Hh (Wnt) signaling pathway was measured by the average phenotype similarity of the gene to all the members in the pathway.

## Authors' contributions

LW, ZT and FS designed the study. LW carried out the computational analysis and validations. LW drafted the manuscript and ZT and FS edited the manuscript. All authors read and approved the final manuscript.

## Supplementary Material

Additional file 1 **Supplementary materials**. This file contains all the supplementary text, tables and figures. Click here for file

Additional file 2 **NePhe scores**. This file contains the NePhe score results for both hits and nonhits of the 24 whole-genome RNAi screens. Click here for file

Additional file 3 **R package NePhe**. This file contains the source codes of the R package for computing NePhe scores. Click here for file
