# Supplementary material for: A network-based integrative approach to prioritize reliable hits from multiple genome-wide RNAi screens in Drosophila
Source: BMC Genomics. 2009 May 12;10:220. doi: 10.1186/1471-2164-10-220 (PMC2697172; doi:10.1186/1471-2164-10-220)
Supplement: Additional file 1 — Supplementary materials. This file contains all the supplementary text, tables and figures. [file 1471-2164-10-220-S1.pdf]

Supplementary Materials

Network connectivity of RNAi hits

Table S1 shows the size of the largest component, the number of isolated nodes and their corresponding P-values for each sub-network derived from each of the 24 RNAi screens. It can be seen that the majority of the 24 sub-networks are characterized by significantly larger connected component and fewer isolated nodes, which indicate higher network connectivity, as compared to random cases (P-values < 0.05). But these two network attributes may not be as powerful as the number of edges in revealing the network connectivity. Since when the number of hits is large enough, e.g., > 300, most hits can be connected in a large connected component even within randomized network, the P-values are hence less significant or not significant for these sub-networks.

Screen-specific Performance of different NePhe scoring functions

We compared the performance of different NePhe scoring functions in each of the 24 RNAi screens. Here we particularly focused on their performance in identifying FPs rather than FNs, since the estimation of the former is far more reliable (with smaller error bars) than the latter in the rank-based tests. Figure S1 shows the screen-specific performance of different methods. It has to be reminded that in the rank-based test, the lower the simulated FPs are ranked among hits, the better the scoring function performs. It can be seen in Figure S1 that the diffusion kernel (DK) based methods (red) generally outperform others. In addition, DK appears to show superiority especially for those screens whose hit sets display certain characteristics. For example, DK shows superiority particularly for hit set of smaller size. DK is the best performed scoring

function for 8 out of the 9 screens with hit size <100. This observation is consistent with previous findings that DK significantly outperforms other similarity measurements in prioritizing human disease genes [1, 2], since the number of known causal genes for a disease is usually within this range. Among those hit sets of larger size, DK shows obvious advantages for relatively "difficult" cases. For instance, DK is the best performed method for "nuclear import of Smads" and "regulators of NFAT". Based on the results of Table 1, the network connectivity of hits for both screens is among the lowest. In contrast, for those well-connected hit sets, such as "viral replication", simple measurements like direct neighbor can achieve the best performance.

Robustness of NePhe scores to random noise in the hit set

We calculated NePhe scores for each of the 24 screens using the best method for that screen according to the rank-based test. We want to know how robust the calculated NePhe scores are to the random noise in the initial hit set. In order to achieve this, we simulated noise in the hit set and then compared the NePhe scores before and after the simulation. More specifically, for each screen, we randomly added a number of nonhits (e.g., 10% of the original hit number) into the original hit set and recalculated the NePhe scores. We then computed Spearman's correlation coefficients for NePhe scores of original hits before and after adding the noise. We repeated the above procedure 20 times and obtained the mean and standard deviation (Table S4). Similarly, we randomly removed a number of hits from the initial hit set and then compared the NePhe scores for the remaining hits before and after the removal (Table S5). As shown in both Table S4 and Table S5, the NePhe scores of the hits appear to be reasonably robust to noise in the initial hit set. Most of the correlation coefficients remain above 0.95, even after adding 50% noise or removing 50% of the original hits. The NePhe scores tend to be less robust for those screens

whose sub-networks show lower connectivity (Table 1). Comparing results in Table S4 and S5, NePhe scores seem to be more robust to the random noise added to the hit set than to the random deletion of original hits. Similar results can be observed for NePhe scores of the nonhits (data not shown).

Sequence-based OTE prediction of FPs

For each dsRNA used to knockdown hits in the original screen, we retrieved the number of 19 nucleotide exact overlaps it has with other genes from flyRNAi database [3]. From the same place, we also obtained the information about whether there are CAN repeats in the dsRNA. Using similar criteria as in the study of DasGupta et al [4], we labeled a hit as off target (OT)-related if its corresponding dsRNA shares greater than 5 possible 19 nt exact overlaps with other genes, or there are CAN repeats in the dsRNA, and labeled it as OT-unrelated if neither the two criteria are satisfied. Those OT-related hits were predicted to be enriched with FPs [5, 6].

Interpret RNAi phenotypes at module level

It is of note that most of the canonical participants of Wnt signaling pathway shown in Figure 6 are clustered together in a single module (module I of Figure 6), so are most of the literature-supported genes (square nodes). This suggests that traditional strategies in identifying participants of Wnt signaling pathway might be biased on certain modules, most likely the core modules [7]. Among the remaining modules that are mainly composed of non-canonical participants, module II is related to transcription factor TFIID complex, including Tbp, e(y)1, Taf1, Taf6 and etc. It is not surprising to observe TFIID complex as regulators of Wnt signaling pathway, since binding of TFIID to DNA is necessary for transcription initiation for most RNA

polymerase II promoters. For example, previous study of the promoter region of mouse frizzled related protein (a family of secreted proteins involved in the Wnt signaling pathway) identified putative transcriptional factor binding sites for TFIID and other transcription factors [8]. Module III is associated with PcG protein complex, a chromatin-associated multi-protein complex containing Polycomb Group proteins, including Z, Pc, ph-p, ph-d and etc. The involvement of PcG complex in Wnt signaling pathway has also been suggested by previous studies. For example, genome-wide binding profiles of Polycomb Group proteins showed that PcG proteins preferentially bound to developmental genes, many of which encode transcriptional regulators and key components of signal transduction pathways, including Wingless, Hedgehog, Notch and Delta [9]. Module IV contains many proteins known to be involved in a diverse of signaling pathways, for instance, Pnt of torso signaling pathway [10], aop of Ras signaling pathway [11], pvr of VEGF receptor signaling pathway [12] and vn of EGF signaling pathway [13]. Their involvement as regulators for Wnt signaling pathway suggests that the complex cross-talking among different signaling pathways could be much more prevailing than previously thought.

Linear regression vs. logistic regression

Both linear and logistic regression have been successfully used for classification problems [14-16]. Thus, both regression models could potentially be adopted in determining the parameters in Formula 2.3. In order to compare the performance of these two strategies in deriving effective NePhe scores, we again employed the rank-based test. Figure 3S plots the screen-specific performance of the two regression strategies in identifying FNs and FPs. As shown in Figure 3S, the two regression strategies are generally comparable to each other, with linear regression slightly better than logistic regression. The superiority of linear regression over logistic

regression is especially obvious when screens with smaller hit size are considered (red in Figure 3S).

References

1. Wu X, Jiang R, Zhang MQ, Li S: **Network-based global inference of human disease genes**. *Mol Syst Biol* 2008, **4**.
2. Köhler S, Bauer S, Horn D, Robinson PN: **Walking the Interactome for Prioritization of Candidate Disease Genes**. *The American Journal of Human Genetics* 2008, **82**(4):949-958.
3. Wilson RJ, Goodman JL, Strelets VB, The FlyBase C: **FlyBase: integration and improvements to query tools**. *Nucl Acids Res* 2008, **36**(suppl_1):D588-593.
4. DasGupta R, Nybakken K, Booker M, Mathey-Prevot B, Gonsalves F, Changkakoty B, Perrimon N: **A case study of the reproducibility of transcriptional reporter cell-based RNAi screens in Drosophila**. *Genome Biology* 2007, **8**(9):R203.
5. Ma Y, Creanga A, Lum L, Beachy PA: **Prevalence of off-target effects in Drosophila RNA interference screens**. *Nature* 2006, **443**(7109):359-363.
6. Kulkarni MM, Booker M, Silver SJ, Friedman A, Hong P, Perrimon N, Mathey-Prevot B: **Evidence of off-target effects associated with long dsRNAs in Drosophila melanogaster cell-based assays**. *Nat Methods* 2006, **3**:833 - 838.
7. Friedman A, Perrimon N: **Genetic Screening for Signal Transduction in the Era of Network Biology**. *Cell* 2007, **128**(2):225-231.
8. Wong VKW, Yam JWP, Hsiao WLW: **Cloning and Characterization of the Promoter Region of the Mouse Frizzled-Related Protein 4 Gene**. *Biological Chemistry* 2003, **384**(8):1147-1154.
9. Tolhuis B, Muijrers I, de Wit E, Teunissen H, Talhout W, van Steensel B, van Lohuizen M: **Genome-wide profiling of PRC1 and PRC2 Polycomb chromatin binding in Drosophila melanogaster**. *Nat Genet* 2006, **38**(6):694-699.
10. Strecker TR, Yip ML, Lipshitz HD: **Zygotic genes that mediate torso receptor tyrosine kinase functions in the Drosophila melanogaster embryo**. *Proceedings of the National Academy of Sciences of the United States of America* 1991, **88**(13):5824-5828.
11. Chang HC, Solomon NM, Wassarman DA, Karim FD, Therrien M, Rubin GM, Wolff T: **phyllopod functions in the fate determination of a subset of photoreceptors in drosophila**. *Cell* 1995, **80**(3):463-472.
12. Cho NK, Keyes L, Johnson E, Heller J, Ryner L, Karim F, Krasnow MA: **Developmental Control of Blood Cell Migration by the Drosophila VEGF Pathway**. 2002, **108**(6):865-876.
13. Schnepp B, Grumbling G, Donaldson T, Simcox A: **Vein is a novel component in the Drosophila epidermal growth factor receptor pathway with similarity to the neuregulins**. *Genes & Development* 1996, **10**(18):2302-2313.
14. Wu B: **Differential gene expression detection and sample classification using penalized linear regression models**. *Bioinformatics* 2006, **22**(4):472-476.
15. Huang X, Pan W: **Linear regression and two-class classification with gene expression data**. *Bioinformatics* 2003, **19**(16):2072-2078.
16. Wuchty S: **Topology and weights in a protein domain interaction network - a novel way to predict protein interactions**. *BMC Genomics* 2006, **7**(1):122.

Figure Legends

Figure S1: Screen-specific performance of different methods in identifying FPs in rank-based test. The notations are the same as in Figure 1. The 24 screens are placed in the same order as in Table 1 (by row), i.e., decreasing order based on the hit size.

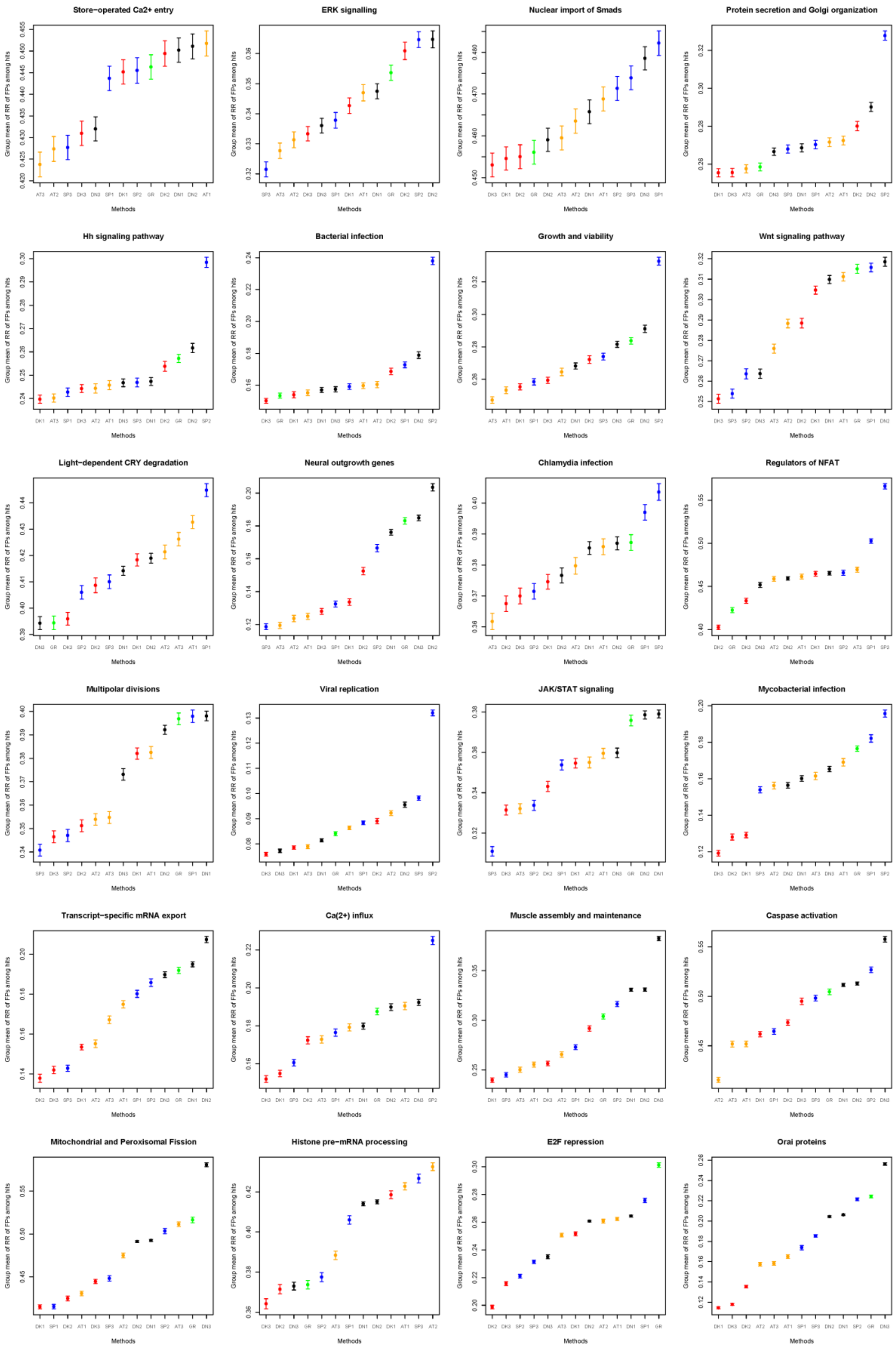
Store−operated Ca2+ entry
ERK signalling
Nuclear import of Smads
Protein secretion and Golgi organization
Hh signaling pathway
Bacterial infection
Growth and viability
Wnt signaling pathway
Light−dependent CRY degradation
Neural outgrowth genes
Chlamydia infection
Regulators of NFAT
Multipolar divisions
Viral replication
JAK/STAT signaling
Mycobacterial infection
Transcript−specific mRNA export
Ca(2+) influx
Muscle assembly and maintenance
Caspase activation
Mitochondrial and Peroxisomal Fission
Histone pre−mRNA processing
E2F repression
Orai proteins
Group mean of RR of FPs among hits
Methods

Figure S2: The reproducibility rate for OT-related and OT-unrelated hits (a), for low-ranked and high-ranked OT-related hits by NePhe score (b) and for low-ranked and high-ranked OT-unrelated hits by NePhe scores (c) for Wnt signaling pathway.

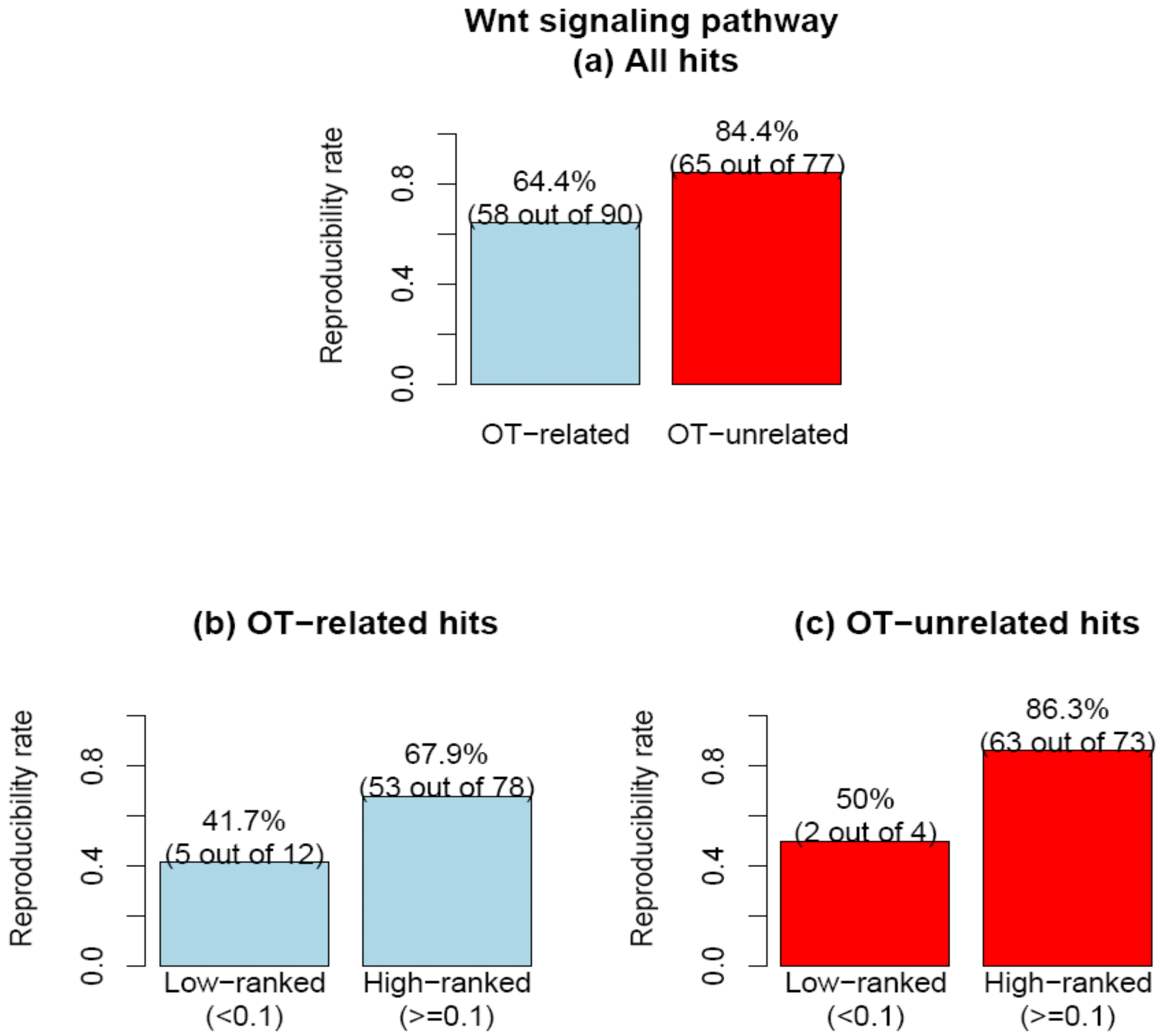

Figure S3: Screen-specific performance of linear regression vs. logistic regression in identifying FNs and FPs in rank-based test. Each point corresponds to one of the 24 RNAi screen. Red points represent screens with hit size <100. The performance is estimated by (a) the group mean of RR of FNs among non-hits (higher means better) and (b) the group mean of RR of FPs among hits (lower means better). The diffusion kernel and Formula 2.3 were used in computing NePhe scores. The diagonal line is drawn to facilitate visual comparison of the performance of the two regression models.

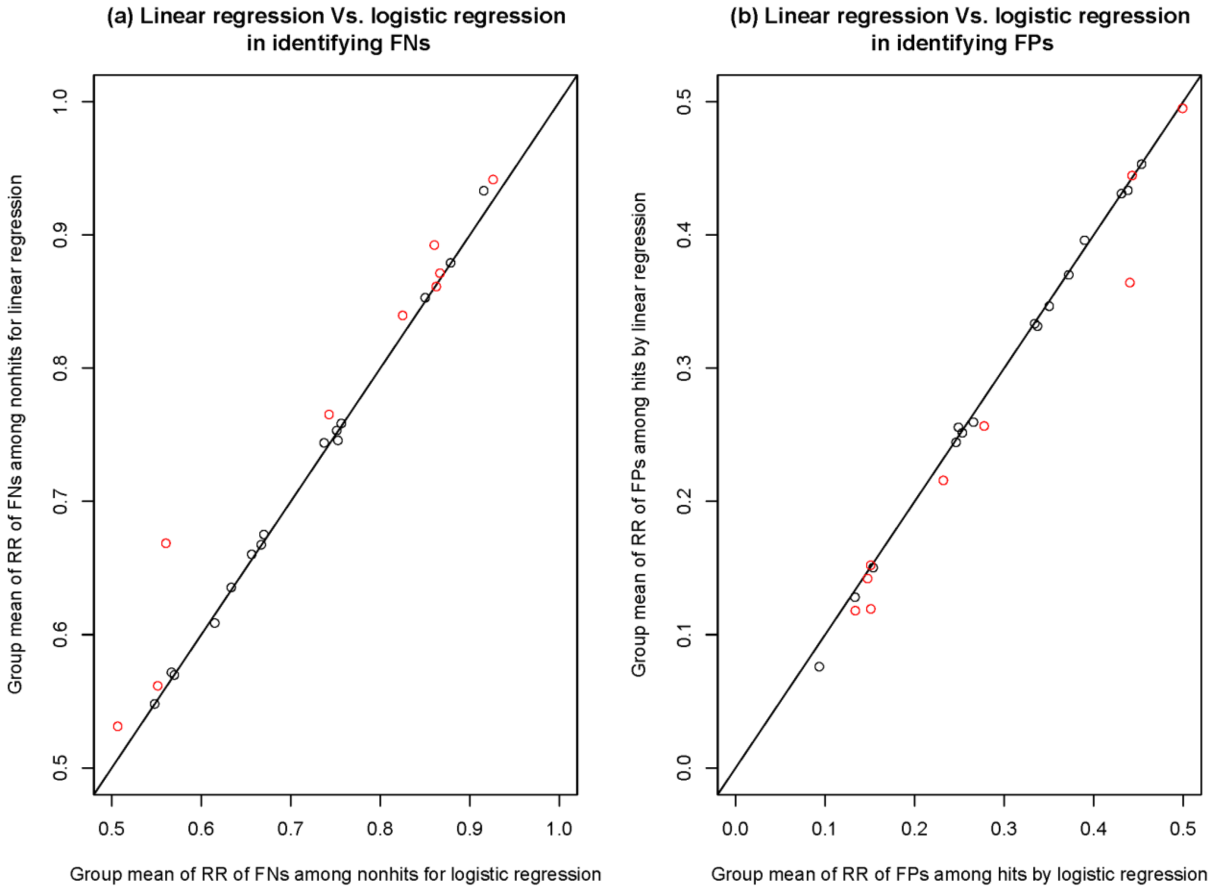

Tables

Table S1: The network attributes and corresponding P-values for the 24 sub-networks constructed from RNAi hits. Results are presented in the same order as in Table 1. The P-value1 and P-value2 are calculated in a similar way to that in Table 1.

RNAi screen	#hits	SLC[†]	P-value1	P-value2	#isolated nodes	P-value1	P-value2
Store-operated Ca2+ entry	1,122	773	6e-05	0.6	326	5e-04	0.1
ERK signaling	982	763	1e-16	1	207	1e-17	1
Nuclear import of Smads	683	377	0.02	1	254	0.004	0.01
Protein secretion and Golgi organization	645	513	3e-16	1	114	8e-26	0.8
Hh signaling pathway	306	230	4e-14	0.8	72	8e-21	0.5
Bacterial infection	286	251	2e-22	0.002	33	2e-35	5e-04
Growth and viability	281	204	8e-14	0.9	72	5e-18	0.6
Wnt signaling pathway	167	101	5e-17	5e-04	61	6e-11	8e-05
Light-dependent CRY degradation	131	39	3e-05	0.4	82	0.03	0.2
Neural outgrowth genes	128	105	2e-54	9e-06	23	5e-23	5e-05
Chlamydia infection	126	55	7e-13	0.004	65	7e-05	4e-04
Regulators of NFAT	121	9	0.6	0.5	83	0.2	2e-04
Multipolar divisions	115	31	3e-05	0.02	63	3e-04	4e-06
Viral replication	104	92	6e-83	0.1	9	5e-31	0.008
JAK/STAT signaling	104	51	6e-23	4e-05	53	1e-05	1e-04
Mycobacterial infection	76	61	2e-99	5e-07	13	7e-22	6e-11
Transcript-specific mRNA export	65	48	2e-86	6e-05	17	3e-17	2e-06
Ca(2+) influx	65	50	2e-94	3e-04	13	8e-21	1e-05
Muscle assembly and maintenance	39	16	6e-28	1e-08	19	4e-07	8e-04
Caspase activation	37	5	0.02	0.01	32	0.4	0.4
Mitochondrial and Peroxisomal Fission	22	3	0.08	0.03	19	0.3	0.2
Histone pre-mRNA processing	17	3	0.02	0.009	12	0.005	0.001
E2F repression	15	7	1e-17	1e-43	6	2e-10	2e-20
Orai proteins	15	4	4e-05	7e-06	4	4e-15	1e-15

† Size of the largest component.

Table S2: The 12 methods for computing NePhe score.

Method	Network-based similarity	Scoring formula
DN1	Direct neighbor (Formula 1.1)	Formula 2.1
DN2	Direct neighbor (Formula 1.1)	Formula 2.2
DN3	Direct neighbor (Formula 1.1)	Formula 2.3
SP1	Shortest path (Formula 1.2)	Formula 2.1
SP2	Shortest path (Formula 1.2)	Formula 2.2
SP3	Shortest path (Formula 1.2)	Formula 2.3
AT1	Association analysis-based transformation (Formula 1.3)	Formula 2.1
AT2	Association analysis-based transformation (Formula 1.3)	Formula 2.2
AT3	Association analysis-based transformation (Formula 1.3)	Formula 2.3
DK1	Diffusion kernel (Formula 1.4)	Formula 2.1
DK2	Diffusion kernel (Formula 1.4)	Formula 2.2
DK3	Diffusion kernel (Formula 1.4)	Formula 2.3

Table S3: The network attributes and corresponding P-values for the 24 sub-networks constructed from RNAi hits after removing those that affect cell growth and viability. Results are presented in the same order as in Table 1. The P-value1 and P-value2 are calculated in a similar way to that in Table 1.

RNAi screen	#hits	#edges	P-value1	P-value2
Store-operated Ca2+ entry	997	2248	0.6	7.00E-14
ERK signaling	862	4151	3.00E-29	3.00E-154
Nuclear import of Smads	662	1163	0.2	2.00E-05
Protein secretion and Golgi organization	421	2212	3.00E-126	<1e-229
Hh signaling pathway	188	672	3.00E-133	4.00E-229
Bacterial infection	190	727	3.00E-158	1.00E-195
Growth and viability	0	NA	NA	NA
Wnt signaling pathway	127	254	1.00E-61	6.00E-80
Light-dependent CRY degradation	118	65	0.004	1.00E-14
Neural outgrowth genes	113	310	6.00E-131	7.00E-117
Chlamydia infection	118	90	1.00E-06	6.00E-14
Regulators of NFAT	119	29	0.6	0.002
Multipolar divisions	109	57	0.004	1.00E-14
Viral replication	55	300	<1e-229	<1e-229
JAK/STAT signaling	86	69	1.00E-10	4.00E-18
Mycobacterial infection	66	165	4.00E-185	5.00E-145
Transcript-specific mRNA export	44	43	2.00E-35	2.00E-31
Ca(2+) influx	51	87	1.00E-100	2.00E-38
Muscle assembly and maintenance	33	7	0.02	0.006
Caspase activation	37	4	0.4	0.2
Mitochondrial and Peroxisomal Fission	22	2	0.3	0.1
Histone pre-mRNA processing	16	4	8.00E-05	2.00E-07
E2F repression	13	6	4.00E-14	3.00E-38
Orai proteins	12	6	2.00E-17	3.00E-23

Table S4: The Spearman's rank correlation coefficients of the NePhe score before and after adding different levels of noise into the hit set. The figure in each cell represents the mean value of the calculated correlation coefficients in 20 simulations. The figure in the parenthesis represents the standard deviation.

RNAi screen	Add 10%	Add 20%	Add 20%	Add 40%	Add 50%
Store-operated Ca2+ entry	0.992(0.002)	0.985(0.003)	0.98(0.004)	0.975(0.004)	0.966(0.009)
ERK signalling	0.999(3e-04)	0.998(4e-04)	0.997(5e-04)	0.996(8e-04)	0.995(0.001)
Nuclear import of Smads	0.97(0.01)	0.943(0.01)	0.918(0.02)	0.89(0.02)	0.867(0.02)
Protein secretion and Golgi organization	0.994(0.003)	0.989(0.002)	0.981(0.004)	0.977(0.004)	0.973(0.004)
Hh signaling pathway	0.999(5e-04)	0.997(0.001)	0.996(0.001)	0.995(0.002)	0.994(0.001)
Bacterial infection	1(2e-04)	0.998(3e-04)	0.998(4e-04)	0.997(6e-04)	0.996(8e-04)
Growth and viability	0.998(0.001)	0.997(0.002)	0.995(0.005)	0.993(0.004)	0.987(0.01)
Wnt signaling pathway	0.994(0.004)	0.989(0.006)	0.983(0.008)	0.977(0.007)	0.97(0.01)
Light-dependent CRY degradation	0.978(0.02)	0.95(0.03)	0.937(0.03)	0.92(0.03)	0.913(0.03)
Neural outgrowth genes	0.997(0.001)	0.993(0.002)	0.99(0.003)	0.988(0.004)	0.985(0.004)
Chlamydia infection	0.992(0.005)	0.983(0.007)	0.975(0.01)	0.973(0.01)	0.957(0.02)
Regulators of NFAT	0.98(0.01)	0.951(0.02)	0.931(0.03)	0.91(0.03)	0.894(0.05)
Multipolar divisions	0.987(0.006)	0.978(0.009)	0.966(0.01)	0.962(0.01)	0.943(0.02)
Viral replication	1(6e-04)	1(3e-04)	1(6e-04)	0.999(7e-04)	0.999(7e-04)
JAK/STAT signaling	0.993(0.002)	0.987(0.005)	0.985(0.003)	0.974(0.02)	0.976(0.007)
Mycobacterial infection	0.996(0.006)	0.991(0.007)	0.982(0.01)	0.972(0.03)	0.976(0.02)
Transcript-specific mRNA export	0.988(0.01)	0.978(0.02)	0.97(0.03)	0.966(0.03)	0.956(0.04)
Ca(2+) influx	0.998(0.002)	0.995(0.004)	0.991(0.005)	0.99(0.004)	0.987(0.006)
Muscle assembly and maintenance	0.993(0.01)	0.975(0.04)	0.95(0.07)	0.955(0.05)	0.94(0.05)
Caspase activation	0.959(0.05)	0.93(0.07)	0.926(0.06)	0.892(0.07)	0.854(0.1)
Mitochondrial and Peroxisomal Fission	0.997(0.01)	0.991(0.01)	0.978(0.05)	0.981(0.02)	0.963(0.03)
Histone pre-mRNA processing	0.966(0.04)	0.925(0.07)	0.897(0.1)	0.88(0.1)	0.91(0.07)
E2F repression	0.998(0.003)	0.992(0.02)	0.991(0.02)	0.998(0.003)	0.992(0.008)
Orai proteins	1(0.001)	0.999(0.002)	0.994(0.01)	0.982(0.04)	0.984(0.04)

Table S5: The Spearman's rank correlation coefficients of the NePhe score before and after removing different fractions of hits from the hit set. The figure in each cell represents the mean value of the calculated correlation coefficients in 20 simulations. The figure in the parenthesis represents the standard deviation.

RNAi screen	Remove 10%	Remove 20%	Remove 20%	Remove 40%	Remove 50%
Store-operated Ca2+ entry	0.991(0.002)	0.983(0.003)	0.972(0.006)	0.96(0.007)	0.951(0.01)
ERK signalling	0.999(2e-04)	0.997(0.001)	0.994(8e-04)	0.99(0.004)	0.986(0.005)
Nuclear import of Smads	0.958(0.01)	0.926(0.02)	0.856(0.09)	0.835(0.03)	0.749(0.09)
Protein secretion and Golgi organization	0.991(0.003)	0.98(0.003)	0.964(0.007)	0.948(0.01)	0.926(0.02)
Hh signaling pathway	0.998(6e-04)	0.996(0.001)	0.994(0.001)	0.99(0.003)	0.988(0.004)
Bacterial infection	0.998(4e-04)	0.995(9e-04)	0.991(0.002)	0.987(0.003)	0.983(0.005)
Growth and viability	0.997(8e-04)	0.996(0.002)	0.99(0.006)	0.988(0.004)	0.984(0.01)
Wnt signaling pathway	0.986(0.005)	0.979(0.01)	0.965(0.01)	0.947(0.02)	0.927(0.02)
Light-dependent CRY degradation	0.984(0.01)	0.953(0.03)	0.942(0.03)	0.921(0.05)	0.882(0.04)
Neural outgrowth genes	0.99(0.004)	0.977(0.006)	0.963(0.007)	0.945(0.01)	0.925(0.02)
Chlamydia infection	0.985(0.01)	0.975(0.01)	0.958(0.02)	0.944(0.03)	0.873(0.2)
Regulators of NFAT	0.924(0.03)	0.838(0.04)	0.782(0.08)	0.706(0.08)	0.626(0.08)
Multipolar divisions	0.983(0.007)	0.962(0.01)	0.938(0.02)	0.89(0.05)	0.824(0.2)
Viral replication	0.998(7e-04)	0.996(0.001)	0.994(0.002)	0.99(0.003)	0.987(0.005)
JAK/STAT signaling	0.989(0.004)	0.978(0.007)	0.968(0.008)	0.933(0.03)	0.913(0.05)
Mycobacterial infection	0.943(0.04)	0.93(0.04)	0.872(0.07)	0.825(0.08)	0.735(0.1)
Transcript-specific mRNA export	0.966(0.03)	0.95(0.02)	0.891(0.06)	0.832(0.07)	0.83(0.1)
Ca(2+) influx	0.981(0.009)	0.958(0.01)	0.943(0.02)	0.914(0.02)	0.863(0.06)
Muscle assembly and maintenance	0.974(0.05)	0.938(0.06)	0.9(0.1)	0.86(0.1)	0.81(0.1)
Caspase activation	0.954(0.03)	0.897(0.06)	0.862(0.08)	0.779(0.1)	0.796(0.1)
Mitochondrial and Peroxisomal Fission	0.994(0.02)	0.982(0.04)	0.954(0.06)	0.943(0.06)	0.91(0.1)
Histone pre-mRNA processing	0.862(0.3)	0.81(0.4)	0.583(0.5)	0.6(0.5)	0.413(0.5)
E2F repression	0.947(0.07)	0.95(0.06)	0.898(0.1)	0.832(0.1)	0.65(0.3)
Orai proteins	0.841(0.2)	0.776(0.2)	0.543(0.3)	0.547(0.3)	0.255(0.4)
